# Road-Traffic Noise: Annoyance, Risk Perception, and Noise Sensitivity in the Finnish Adult Population

**DOI:** 10.3390/ijerph120605712

**Published:** 2015-05-26

**Authors:** Enembe Oku Okokon, Anu W. Turunen, Sari Ung-Lanki, Anna-Kaisa Vartiainen, Pekka Tiittanen, Timo Lanki

**Affiliations:** Department of Health Protection, National Institute for Health and Welfare, Neulaniementie 4, P.O. Box 95, FI-70701 Kuopio, Finland; E-Mails: anu.turunen@thl.fi (A.W.T.); sari.ung-lanki@thl.fi (S.U.-L.); anna-kaisa.vartiainen@thl.fi (A.-K.V.); pekka.tiittanen@thl.fi (P.T.); timo.lanki@thl.fi (T.L.)

**Keywords:** road-traffic noise, noise annoyance, noise sensitivity, road-traffic exhaust, air pollution, environmental noise

## Abstract

Exposure to road-traffic noise commonly engenders annoyance, the extent of which is determined by factors not fully understood. Our aim was to estimate the prevalence and determinants of road-traffic noise annoyance and noise sensitivity in the Finnish adult population, while comparing the perceptions of road-traffic noise to exhausts as environmental health problems. Using a questionnaire that yielded responses from 1112 randomly selected adult Finnish respondents, we estimated road-traffic noise- and exhausts-related perceived exposures, health-risk perceptions, and self-reported annoyance on five-point scales, while noise sensitivity estimates were based on four questions. Determinants of noise annoyance and sensitivity were investigated using multivariate binary logistic regression and linear regression models, respectively. High or extreme noise annoyance was reported by 17% of respondents. Noise sensitivity scores approximated a Gaussian distribution. Road-traffic noise and exhausts were, respectively, considered high or extreme population-health risks by 22% and 27% of respondents. Knowledge of health risks from traffic noise, OR: 2.04 (1.09–3.82) and noise sensitivity, OR: 1.07 (1.00–1.14) were positively associated with annoyance. Knowledge of health risks (*p* < 0.045) and positive environmental attitudes (*p* < 000) were associated with higher noise sensitivity. Age and sex were associated with annoyance and sensitivity only in bivariate models. A considerable proportion of Finnish adults are highly annoyed by road-traffic noise, and perceive it to be a significant health risk, almost comparable to traffic exhausts. There is no distinct noise-sensitive population subgroup. Knowledge of health risks of road-traffic noise, and attitudinal variables are associated with noise annoyance and sensitivity.

## 1. Introduction

Road-traffic noise is a widespread environmental nuisance which affects people in their residential dwellings and workplaces [1]. It interferes with human ability to function optimally in daily life [2,3,4] and is rated as the most important source of community noise [5,6]. A WHO report estimates that about 65% of the population in the European Union resides in places where they are regularly exposed to noise levels ranging from 55 to 65 dB. Much of this noise is road traffic related. Exposure of this magnitude has been associated with stress reactions, sleep disturbances and poor health [7,8,9,10]. Of further concern are links which have been shown to exist between sustained noise exposure and an increased mortality risk [4,11,12,13,14]. A common human reaction to noise is annoyance. Noise annoyance can be seen as a negative emotional and attitudinal reaction to noise. The concept of noise annoyance is, however, not unambiguous [15], even though an ISO standard has been created for the estimation of annoyance [16].

Noise annoyance is thought to be an indicator of environmental wellbeing, and is sometimes considered to be a harbinger of subsequent health effects engendered by noise [8]. Although annoyance is a subjective response to noise exposure, it is not explained entirely by ambient acoustics [17]. Rather, it is subject to the influence of personal traits, demographic characteristics, and also physical attributes of the environment which collectively modify the complex psychophysiological sequence leading from noise exposure to annoyance [18]. Interventions designed to reduce noise nuisance should take into account subject-specific determinants of annoyance, as this could increase citizens’ support for such initiatives, and consequently, increase success rate.

Individual attributes observed to influence noise annoyance include: age, sex, marital status, [19,20], having children, level of education [21] and occupational status, for example [22]. Age and sex have been widely investigated as determinants of noise annoyance, but frequently conflicting results are reported [8]. Nonetheless, these variables are relevant as proxies for other factors that modify the individual’s perception of and response to environmental stimuli. Subjective appraisal of environmental quality has been shown to influence human perception of noise and consequent annoyance [23]. Relatively few studies have thus far explored the relationship between attitudinal factors and noise annoyance. For some time has been known that persons who pay closer attention to their immediate environment have a tendency to be more disturbed by noise intrusion, suggesting a proneness to attentional processes [24]. It is not known, however, if this tendency can be generalised to people who care for the environment in general (people having positive environmental attitudes) or those who prefer nature. These are attributes which are subsets of an individual’s psychosocial construct, which evidently mediate subjective response to environmental stimuli [9].

Relating to the physical environment, it must be stressed that although the residential milieu is for most people distinct from the occupational environment, the individual who traverses both settings may ‘import’ stress from one place to another. For example, it has been suggested that noise at work likely modifies the individual’s attitude to noise in general by increasing susceptibility to negative noise response [5]. It may be that one who endures noise exposure at work longs for an escape at the cessation of work, but further noise encounter at home escalates incipient frustration and annoyance.

Noise sensitivity is the singular personal attribute which demonstrably is the most consistent predictor of noise annoyance [5,25]. Noise sensitive persons are known to be more subject to noise annoyance than non-sensitive persons. Furthermore, noise sensitivity has been suggested to predict noise-induced health effects [26]. Despite the association between this trait and noise annoyance, variations in noise sensitivity have no established relationship with the extent of noise exposure [25,27]. Paradoxically, some studies have proposed that prolonged noise exposure may heighten noise sensitivity [25,28].

Noise sensitivity is further described as an intrinsic personal trait having an affective dimension [29]. Meijer *et al.* [23], and Ryu and Jeon [30] argue that it may be an indicator of general sensitivity to poor environmental quality; Stansfeld has reported a relationship between noise sensitivity, anxiety and depression [31]. Of further note is the observation that noise sensitive persons could exhibit negative affectivity toward self and a broad range of environmental factors in a manner not unlike trait anxiety [31,32]. In contrast, Schreckenberg *et al.* maintains that noise sensitivity is related to physical rather than mental health states. Schreckenberg *et al.* [24] and Miedema and Vos [27] separately frame noise sensitivity as a more discriminating sensitivity toward sound quality and residential environmental safety rather than being a broad negative attitude toward the general environment.

Studies exploring the relationship between noise levels and noise annoyance have traditionally used measured- or modelled-sound levels. This approach has limited application in wide-scale national surveys which involve participants recruited from urban and rural areas because noise maps exist mostly for cities, and the cost of instrumental monitoring in multiple non-contiguous rural settings could be daunting. Another consideration is that between the acoustic parameters of emitted sound and what the human ear perceives as noise are several personal and environmental factors which remain only partially understood [33,34].

This study applied a questionnaire assessment of perceived road-traffic noise and air pollution to explore the complex relationship between exposures and human response in adults living in Finland: a northern European country with a low population density estimated at 16 persons per square kilometer [35]. Road-traffic noise and exhausts are investigated together because they are co-stressors which arise from a shared ubiquitous source, and from a public well-being perspective, it is important to know which of these is considered more important by the public. It is also pertinent to know if the discomfort caused by these stressors accumulate on certain individuals, and what factors identify these individuals. The survey evaluated self-reported noise and air pollution exposures, annoyance, perceived health risks from exposures, noise sensitivity, and the knowledge and attitudes pertaining to these exposures, with the following aims: (1) to determine the prevalence of road-traffic related noise annoyance and noise sensitivity; (2) to identify and quantify factors associated with road-traffic noise annoyance, and noise sensitivity; (3) to compare the perceptions of road-traffic noise and air pollution as environmental health problems.

## 2. Methodology

This study was survey based and involved the self-administration of a questionnaire by Finnish adults.

### 2.1. Study Sample

A simple random sample of 3000 Finnish-speaking persons living in mainland Finland (excluding Åland), aged between 25 and 74 years, was obtained from the Population Register Centre (2011). Data collection was conducted in the autumn of 2011 by postal dissemination of the study questionnaire. After one round of written reminders, 1112 respondents returned the questionnaire yielding a response rate of 37% (43.9% male and 56.1% female). The data well represents the socio-demographic structure of the target population. Deviations from the target population are: <1% based on region, and 0.2% to 4.7% based on vocational status. Women (deviation from the target population being 5.8%) and older people (deviation from the target population in different age groups of 0.5%–4.6%) were slightly overrepresented.

### 2.2. Study Variables

We used a semi-structured questionnaire constructed to assess road-traffic noise and road-traffic exhaust related parameters such as perceived exposure, annoyance, perceptions and knowledge of health risks, noise sensitivity, concerns about health risks from home and occupational environments, and environmental attitudes. (Table A1). Additionally, the instrument had entries for respondents’ demographic data. We focused on demographics which, according to the literature, modify noise annoyance, such as: age, sex, marital status, presence of children in the family, residential area, vocational education and other demographic variables. The questionnaire was not supplemented by noise maps or instrumental measurements because of the impracticality of implementing such measurements across the broad geographical spread of respondents—who were sampled across Finland, noting that Finns are known to live far apart outside cities. Noise maps are mostly available for cities, but rural areas, such as we drew some of our respondents from, are poorly covered by these maps.

Perceived exposures were determined with questionnaire items 1–4 with responses rated on a five-point scale. Annoyance due to noise or exhaust exposure was also assessed on a five-point scale using questionnaire items 6–7. Symptoms developed from either noise or exhaust exposures were assessed with questionnaire items 7–8. Subjective estimation of health risks from environmental stressors was assessed with questionnaire items 9–12 (Table A1). Composite measures were calculated for: environmental attitudes (items 16–18), based on questions that were adopted from the 2010 International Social Survey Programme questionnaire [36]; nature orientedness (items 19–25), based on a scale developed by Korpela *et al.* [37]; and noise sensitivity (items 26–29) which was based on four items excerpted from Weinstein’s 21-item noise sensitivity scale [38]. Composite scores for environmental attitudes were 0 to 12 with lower scores representing positive environmental attitudes and vice versa; sum scores for nature orientedness ranged from 0 to 28 with higher scores representing preference for nature while lower scores denote preference for built-up areas. Noise sensitivity composite scores ranged from 0 to 16 with higher scores denoting higher sensitivity.

We reclassed persons who neither reported residential nor workplace traffic-noise (or exhausts) exposures as not experiencing annoyance or symptoms: regardless of their responses to the pertinent question. This is because these variable were framed to elicit responses to ‘felt’ pressures from exposures. Also, persons reporting no exposures at home, and who were not working during the period of data collection, were reclassed as neither annoyed nor having symptom.

For statistical modelling of covariates of noise annoyance, we collapsed annoyance ratings into dichotomous groups by combining the answers high and extreme (score points 4 and 5) as annoyed and the other categories as not annoyed; this was done to facilitate an increased statistical power of our model to identify covariates of high to extreme noise annoyance. We recoded the predictors of noise annoyance (perceptions of residential traffic noise exposure; perceptions of occupational traffic noise exposure; worry about health risk to self and family arising from the home environment; worry about health risks to self arising from the occupational environment; and knowledge of health risks associated with traffic noise into three categories) viz. score points 1 and 2 = category 1, score points 3 = category 2 and score points 4 and 5 = category 3.

For modelling of noise sensitivity, noise sensitivity was inserted into the model as a continuous variable. we kept the covariates of noise sensitivity in their original scales, specifically, a five-point scale for discrete variables that were elicited in this manner, a dichotomous scale for variables such as sex and hearing impairment, or the actual numeric values for continuous variables (e.g., age). For convenience of statistical analyses, we also recoded respondents’ occupational groupings from the original 9 categories into 5 categories: executive employee/upper clerical worker = category 1, lower clerical worker/employee = category 2, entrepreneur or self-employed/agricultural entrepreneur or farmer = category 3, pensioner = category 4 and student/homemaker/unemployed/others= category 5).

### 2.3. Statistical Analyses

We used the binary logistic regression analysis to estimate which factors were associated with noise annoyance. Logistic regression models the odds of the probability of an event occurring on a logarithmic scale. This transformation (log odds) allows an approximation of the linear regression model for a binary variable. We used a multivariate linear regression analysis to estimate covariates of noise sensitivity.

In building the multivariate models, we first checked for association between predictor variables and outcome variables using bivariate binary logistic regression and bivariate linear regression for noise annoyance and noise sensitivity, respectively. Pre-tested predictor variables were: sex, age, education, occupational status, having children, residential road-traffic noise exposures, occupational road-traffic noise exposures, symptomatic reactions to road-traffic noise exposure, personal worry about environmental health risks to self and family arising from the residential environment, personal worry about environmental health risks to self arising from the occupational environment, self-rated personal knowledge of health risks associated with road-traffic noise, subjective assessment of personal health, hearing impairment, environmental attitudes, nature orientedness, noise sensitivity (in the noise annoyance prediction model) and noise annoyance (in the noise sensitivity prediction model).

We simultaneously incorporated all predictors which yielded statistically significant results from bivariate models into the multivariate models. At successive iterations, single predictors which did not yield statistically significant association, and which additionally had the largest *p*-values were eliminated from the model leaving only predictors with *p*-values < 0.2 in the final output. *Post hoc*, we assessed for differences in the expressions of covariates of noise annoyance and noise sensitivity between sexes using a cross-tabulation technique for categorical variables and the Wilcoxon Rank Sum test for the attitudinal variables.

We tested for multi-collinearity between noise annoyance, noise sensitivity, environmental attitudes and nature orientedness. Although these variables were correlated (with statistical significance), multicollinearity was minimal in all cases. We discovered a high statistical correlation between noise annoyance and development of symptoms from traffic-noise exposures, worry about health risks from the residential environment, worry about health risks from the occupational environment. We believe that these variables measured the same underlying construct; therefore, we decided to exclude ‘symptom development’ and ‘worry about health risks arising from the occupational environment’ from the model building process.

We used the Wilcoxon Signed-Rank test to determine if persons reporting higher (high to extreme categories) exposure to road-traffic noise or exhausts at home would report similar exposure at work. Similarly, we compared symptom perception from exposure to road-traffic noise and exhaust exposures. We explored the relationship between road-traffic related noise annoyance and road-traffic exhausts using Spearman’s Rank Order correlation coefficient. We used this same method for all correlation analyses. All analyses were performed using IBM SPSS Statistics version 22 (IBM Corp., Armonk, NY, USA) and with a 0.05 level of significance.

## 3. Results

In sum, 1112 persons returned the questionnaire which implies a response rate of 37%. The mean age of the respondents was 53.7 ± 13.4 years (range: 25–75 years), and 40% of these were elderly persons (aged 60–74 years). The majority of the respondents were females (56%), and 63% of all respondents were working at the time of the survey (Table 1).

**Table 1:** Demographic data of respondents.

Variable	Categories	% (Number)
Sex	Males	43.9 (488)
	Females	56.1 (624)
Age group	25–44	26.0 (289)
	45–59	33.9 (377)
	60–74	40.1 (446)
Marital status	Single	13.2 (146)
	Married or in a registered relationship, cohabiting	74.1 (820)
	Divorced or separated or widowed	12.8 (141)
Occupational status	Executive employee, upper clerical worker	17.8 (197)
	Lower clerical worker, employee	35.2 (390)
	Entrepreneur, self-employed, agricultural entrepreneur, farmer	8.9 (99)
	Pensioner	29.5 (327)
	Student/Homemaker/unemployed/others	8.7 (96)
Children in the family	No	22.4 (246)
	Yes	77.6 (853)
Residential area	Downtown city centre	13.1 (145)
	City suburb	48.5 (537)
	Population centre in the countryside	17.4 (193)
	Sparsely populated area	21.0 (232)
Vocational education	No vocational training, professional course, other short vocational training	23.1 (253)
	Vocational school, school level vocational examination, college level vocational examination	46.6 (509)
	Higher vocational diploma, University degree	30.4 (332)

### 3.1. Prevalence of Road Traffic-Noise and Exhaust Exposures

In total, 80% of the respondents reported some level of residential exposure to road-traffic noise, and 18% reported high to extreme exposure (score points 4 and 5). Similarly, 13% of respondents reported high to extreme residential exposure to traffic exhausts. The prevalence of high to extreme traffic noise exposure was higher at home than in the workplace (*p* < 0.001) (Table 2).

On selectively comparing home *versus* workplace ratings of road-traffic noise only for persons reporting high to extreme exposures, there was a statistically significant difference (*p* < 0.020). Disparately, equivalent ratings were seen on comparing home *versus* workplace ratings of road-traffic exhaust exposure (*p* < 0.504).

**Table 2:** Self-rated exposures to road traffic related noise and air pollution.

Exposure Variable	1 = No Exposure	2	3	4	5 = Extreme Exposure	Total
	% (Number)	% (Number)	% (Number)	% (Number)	% (Number)	% (Number)
Perceived Residential Exposure (*n* = 1112)						
Traffic noise	19.8 (216)	37.7 (411)	24.7 (270)	12.9 (141)	4.9 (53)	100.0 (1091)
Traffic exhaust	17.0 (184)	39.1 (423)	30.9 (334)	10.2 (110)	2.8 (30)	100.0 (1081)
Perceived Workplace Exposure (*n* = 698)						
Traffic noise	30.8 (207)	31.0 (208)	20.9 (140)	12.7 (85)	4.6 (31)	100.0 (671)
Traffic exhaust	29.0 (195)	33.9 (228)	22.7 (153)	9.8 (66)	4.6 (31)	100.0 (673)

### 3.2. Subjective Reaction to Road-Traffic Noise and Exhausts Exposures

Many respondents (65%) reported some (any) annoyance from traffic noise, about 17% reported high to extreme annoyance. About 34% of downtown/city dwellers and 21% of city suburb dwellers reported high to extreme noise annoyance. Road-traffic noise annoyance correlated well with traffic-exhaust annoyance (rho = 0.6). Selectively comparing symptom ratings only for respondents reporting high to extreme symptomatic reaction to either road-traffic noise or road-traffic exhausts yielded no difference (*p* < 0.671).

**Figure 1:**
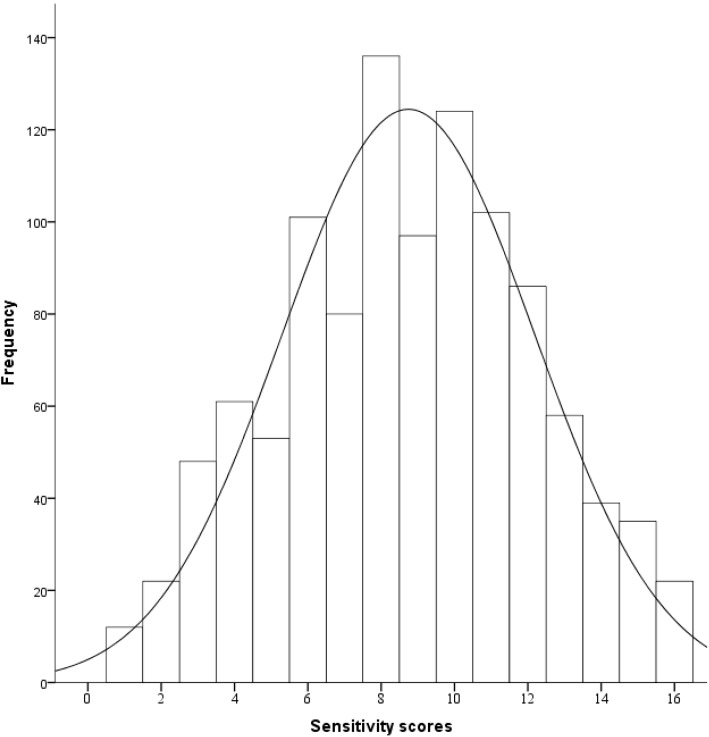
Distribution of noise sensitivity scores in the study population (the solid line indicates the Gaussian distribution).

A considerable proportion of people, 22%, perceived that noise is a population-health risk of high to extreme grade. However, a higher percentage (27%) of people perceived road-traffic exhausts as being of high or extreme risk to the health of the general population. Overall, more respondents perceived that they were at some (personal) health risk from road-traffic exhausts than from noise (*p* < 0.001); but, the proportion of respondents reporting high to extreme risk from both road-traffic noise and exhaust were comparable. More subjects perceived that general population-health risk from traffic noise was higher when compared to their individual-health risk (*p* < 0.001). This finding was similar for perceived risk from traffic exhausts (Table 3). The median noise sensitivity (composite) score was 9 (interquartile range: 6–11), and the distribution approximated a Gaussian distribution. (Figure 1). Self-ratings of noise sensitivity in response to the single question (questionnaire item number 26, Appendix) showed that 35% and 11% of study respondents considered themselves to be highly or extremely noise sensitive, respectively.

**Table 3:** Self-rated feelings relating to road-traffic noise and exhaust exposures.

Exposure Variable	1 = None	2	3	4	5 = Extreme	Total
	% (Number)	% (Number)	% (Number)	% (Number)	% (Number)	% (Number)
Personal Annoyance ^a^						
Traffic noise	34.9 (388)	29.0 (323)	18.9 (210)	12.3 (137)	4.9 (54)	100.0 (1112)
Traffic exhaust	39.9 (444)	36.1 (401)	14.7 (163)	6.9 (77)	2.4 (27)	100.0 (1112)
Develops Symptoms from Exposure						
Traffic noise	63.5 (706)	19.0 (211)	10.0 (111)	5.4 (60)	2.2 (24)	100.0 (1112)
Traffic exhaust	65.1 (724)	18.3 (204)	9.2 (102)	4.9 (55)	2.4 (27)	100.0 (912)
Perception of Personal Health Risk						
Traffic noise	44.5 (430)	30.0 (290)	15.4 (149)	7.6 (73)	2.6 (25)	100.0 (967)
Traffic exhaust	29.0 (283)	36.3 (354)	23.5 (229)	7.0 (68)	4.3 (42)	100.0 (976)
Perception of Population Health Risk						
Traffic noise	7.7 (79)	32.7 (337)	37.3 (384)	17.3 (178)	5.0 (52)	100.0 (1030)
Traffic exhaust	3.6 (38)	28.9 (303)	40.9 (429)	19.5 (205)	7.1 (75)	100.0 (1050)
Noise Sensitivity ^b^						
Noise sensitivity	13.5 (149)	25.7 (283)	25.5 (280)	24.7 (272)	10.5 (116)	100.0 (1100)

^a^ Traffic noise annoyance was calculated only for respondents who felt they were exposed either at home or at work or both; ^b^ Noise sensitivity was calculated for all respondents who provided self-estimate by answering the single question, “*Are you noise sensitive?*”.

### 3.3. Determinants of Noise Annoyance

Covariates which were statistically significantly associated with noise annoyance in the bivariate model were sex (women showed more annoyance to noise, *p* < 0.047), perception of residential exposure to traffic noise, perception of workplace exposure to traffic noise, worry about health risks to self and family arising from the home environment, worry about health risks to self arising from the occupational environment, knowledge of health risks associated with traffic noise, hearing impairment, environmental attitudes, nature orientedness and noise sensitivity.

The multivariate model showed that increased annoyance was positively associated with: self-rated home exposures, personal worry about risks from home environment, and knowledge of health risks due to traffic noise (Table 4). Noise sensitivity and nature orientedness showed borderline significant associations with road-traffic noise annoyance. Nagelkerke R-squared for this model was 0.54, indicating a reasonable explanatory power for the outcome. The distribution of these variables between the two levels of annoyance is shown in Table A2.

**Table 4:** Determinants of high to extreme noise annoyance.

Parameter	Response Category	Odds Ratio	95% C.I. of Odds Ratios	
			Lower	Upper
Perceived exposure to traffic noise at home	No exposure	ref.		
	Some exposure	3.78	2.02	7.06
	Extreme exposure	56.94	30.81	105.23
Worry about health risk from residential environment	Not worried	ref.		
	Worried	2.15	1.27	3.64
	Extremely worried	2.89	1.56	5.37
Knowledge about health risk from traffic noise	no knowledge	ref.		
	some knowledge	1.24	0.69	2.22
	much knowledge	2.04	1.09	3.82
Noise sensitivity ^a^	-	1.07	1.00	1.14
Nature orientedness ^b^	-	1.05	1.00	1.10

^a^ Noise sensitivity was rated on a scale of 0–16; ^b^ Rated on a scale of 0–28, with higher scores indicating love for nature while lower scores signify love for built-up areas.

We carried out a sensitivity analysis (not shown) for the binary logistic model using score points three and above to determine covariates of moderate to extreme noise annoyance. Covariates similar to those found in the first model (excluding nature orientedness) remained after step-wise elimination; in addition, environmental attitudes was also retained in this model (Nagelkerke R^2^ = 0.53). Outcome estimates in this model were: (1) perceived residential exposures to traffic noise—“some exposure” OR 9.89 (6.66–14.67), “extreme exposure”, OR 52.72 (29.09–95.56); (2) worry about risk from residential environment-“worried” OR 1.18 (0.77–1.79), “extremely worried” OR 1.83 (1.06–3.15); (3) knowledge about risk from traffic noise exposure—“some knowledge” OR 1.30 (0.83–2.04), “much knowledge” OR 1.85 (1.12–3.07); noise sensitivity OR 1.09 (1.03–1.15); environmental attitudes OR 0.96 (0.89–1.03).

### 3.4. Determinants of Noise Sensitivity

Factors which showed statistically significant associations with noise sensitivity in the bivariate model were sex (women were more sensitive to noise, *p* < 0.008), occupational status (the occupational category consisting of entrepreneurs, self-employed and farmers was more sensitive), perceptions of residential exposure to traffic noise, traffic-noise annoyance, symptomatic reaction following traffic-noise exposure, worry about health risk to self and family arising from the home environment, worry about health risk to self and from the occupational environment, knowledge of health risks associated with traffic noise (higher knowledge was associated with higher sensitivity), environmental attitudes, nature orientedness.

The multivariate linear regression model showed evidence of statistical associations between noise sensitivity and the following covariates: vocational status, knowledge of health risk from residential noise exposure, positive environmental attitudes, and nature orientedness (respondents with positive environmental attitudes or preference for nature were more noise sensitive). The model accounted for approximately 17% of the variability in noise sensitivity and the overall predictive capacity was statistically significant (F = 12,628, *p* < 0.001) (Table 5).

**Table 5:** Determinants of noise sensitivity.

Parameter	Response Category	β	95% C.I. of Coefficients ^a^		*p*-Value ^b^
			Lower Bound	Upper Bound	
Intercept		9.85	8.19	11.50	0.000
Occupational status	Student, homemaker, unemployed, others worker	Ref.			0.026
	Pensioner	−0.19	−1.02	0.65	
	Entrepreneur, self-employed, agricultural entrepreneur, farmer	−1.26	−2.27	−0.25	
	Lower clerical worker, employee	−0.79	−1.58	0.01	
	Executive employee, upper clerical	−0.75	−1.61	0.11	
Traffic noise annoyance	5 = extremely annoyed	Ref.			0.000
	4	0.77	−0.35	1.89	
	3	0.42	−0.65	1.48	
	2	−0.12	−1.16	0.91	
	1 = not annoyed	−1.53	−2.59	−0.47	
Knowledge of health risks from traffic noise	5 = extremely knowledgeable	Ref.			0.045
	4	−1.11	−2.20	−0.03	
	3	−1.26	−2.31	−0.22	
	2	−1.65	−2.74	−0.57	
	1 = not knowledgeable	−1.53	−3.17	0.11	
Environmental attitudes ^c^	-	−0.23	−0.31	−0.15	0.000
Nature orientedness ^d^	-	0.12	0.08	0.16	0.000

^a^ Linear regression coefficient; ^b^ *P*-values are based on between-subject difference for each variable; ^c^ Rated on a scale of 0 to 12; persons with lower scores had positive environmental attitudes and the converse holds for negative environmental attitudes; ^d^ Rated on a scale of 0–28, with higher scores indicating preference for nature while lower scores signify love for built-up areas.

The *post hoc* exploration of covariates of noise annoyance and noise sensitivity by sex showed statistically significant differences between men and women for all covariates except for nature orientedness. Women tended to be more knowledgeable about risk from traffic noise (χ^2^ = 8.18, *p* < 0.17), more worried personal and family risk arising from the residential environment (χ^2^ = 31.2, *p* < 0.001), and had more positive environmental attitudes (*p* < 0.001).

## 4. Discussion

We conducted a survey to assess perceived road-traffic noise and exhaust exposure, risk perception, and measures of subjective response to exposures. Our results show that more (17%) respondents considered themselves exposed to high or extreme levels of residential road-traffic related noise than high or extreme levels of road-traffic exhaust (13%). Perception of high to extreme health risks was similar for both road-traffic noise and road-traffic exhaust. The distribution of noise sensitivity within the study population approximated a Gaussian distribution. High to extreme noise annoyance was associated with high perceived exposure, increased noise sensitivity, increased worry about environmental safety, greater knowledge of traffic noise health risks and nature orientedness. High to extreme road-traffic noise sensitivity was associated with increased traffic noise annoyance, higher knowledge of health risks from traffic-noise, positive environmental attitudes and nature orientedness. Much of the variability in noise sensitivity remained unexplained.

### 4.1. Perceived Traffic Noise and Exhaust Exposures and Subjective Response

Noise perception draws upon physical characteristics of noise, the immediate disposition of an exposed subject and his attitudes which are determined by underlying personal traits [3]. It is a closer representation of the perceptual quality of noise to a hearer than loudness, and a better predictor of noise-related health outcomes [3]. Comparisons between objective sound measures and noise perception show some agreement, but this has been in so complex a manner as cannot be easily predicted or modelled [7]. Subjective assessments of noise exposure have been previously used mostly in occupational surveys. Neitzel used questionnaire items to assess the perceived intensity of noise in three different work-noise (continuous, intermittent and highly variable) environments. Responses to questions framed to evaluate the absolute loudness of noise and relative loudness were then analysed for relationship with time-logged L_eq_ and L_avg_ readings; their results showed that the survey response categories correlated with measured noise levels comparably to or slightly better than an exposure assignment based on job title. The survey instrument could reasonably contrast between noise levels and between degrees of noise variability [39]. An earlier study among patients with acoustic neuroma and their controls showed that in both groups, there was good agreement between self-reported occupational noise exposure and a job-exposure matrix, with study participants showing a clear distinction of occupations subject to noise levels of 80 dB(A) and above—a limit at which regulations require use of personal protective devices [40]. Dzhambov and Dimitrova, in a community survey, compared a subjective noise exposure, measured on a visual analogue scale, with residential L_den_ and found a high correlation between both metrics [41]. The investigators developed the instrument through rigorous qualitative and quantitative phases, and it is noteworthy that the instrument could predict annoyance, and like measured noise it had no association with noise sensitivity. Although several of these studies have used modified questionnaire items from prior studies, there is presently no broad consensus on the content of survey instruments.

Heinonen-Gujezev *et al.* [42] compared self-reported noise exposure to a map-predicted (modelled) noise exposure. Dichotomized scales for both measures agreed with each other, but the agreement was more for aircraft noise than either railway or road-traffic noise. Aydin observed different patterns of correlation between modelled noise emanating from Frankfurt airport and perceived noise by residents living east or west of the airport with distinct exposure levels [7]. We found that a considerable proportion of Finnish adults reported high to extreme levels of perceived residential road-traffic noise. This underscores the fact that road traffic noise is not a problem that is limited to only densely populated countries with commensurate dense vehicular traffic. More respondents felt they received higher exposure to both road-traffic noise and exhaust emissions at home than at work. Persons reporting high to extreme exposures to road-traffic noise at home were distinct from those reporting same exposure levels at work. On the other hand, persons reporting high to extreme residential traffic exhaust exposures also reported similar occupational road-traffic exhaust exposure. Subjective rating of high to extreme exposure was higher for road-traffic noise than road-traffic exhaust exposures. A similar perceptive pattern was reported in a survey which was conducted in Edinburgh [43].

In Finnish adults, we found a population prevalence of 17% for high to extreme levels of noise annoyance. Other studies which used similar five-point scales have reported higher prevalence for these upper-end responses. For example, a prevalence of 31.4% for “very annoyed” and “extremely annoyed” was obtained in a survey that was conducted in Belgrade [44], and an Egyptian study yielded 65.3% prevalence for similar categories of noise annoyance [45]. The higher prevalence may be explained by differences in traffic composition, population size and transportation policy. Belgrade is reported to have a high number heavy duty vehicles moving both during the day and at night [44], Cairo has a large population and heavier traffic load [45]. Finland on the other hand has an infrastructure that supports bicycle use as a common means of transportation all the year round. A pertinent difference between this study and others, which may account the lower prevalence of annoyance, is the fact that respondents from the countryside were included—other studies were city based.

An experience of high or extreme annoyance from noise exposure was higher than due to traffic exhausts. However, approximately equal numbers of persons reported symptoms from either road-traffic noise or road-traffic exhausts. Additionally, people who reported high to extreme symptoms from road-traffic noise were more likely to report high to extreme symptoms from road-traffic exhausts. Although fewer persons reported exposure to exhaust emissions, the proportion of persons who experienced symptoms from this pollutant was comparable to noise, suggesting a higher potency of traffic exhaust to induce symptoms.

We also observed that respondents estimated that they perceived higher personal and population health risk due to traffic exhausts exposure than from traffic noise. This may explain their higher perception of bodily symptom due to exhaust exposure. A similar pattern of health risk perception was documented in another study, which appraised respondents’ ratings of overall community levels of pollutants, in which air pollution was rated to be the most offensive environmental stressor followed by community noise [44]. In our study, perceptions of risks to the general population, from these stressors, were always higher than perceptions of personal risk. Although population perception of health risk from road-traffic noise receives very scant mention in literature, it may provide an insight into how annoyance and symptoms due to noise can vary in a population.

### 4.2. Factors Associated with Noise Annoyance

We found that self-rated ‘high to extreme’ noise annoyance was influenced by perceived exposures. An enquiry into the relationship between residential (measured) noise levels and subjective noise perception, as well as their influence on noise annoyance has been made by Heinonen-Gujezev *et al.* [42]. The authors used a single item self-rated question to assess noise exposure, and a 10-item question, mostly on disturbance parameters, to estimate noise annoyance. A positive association was seen between increasing noise exposure and annoyance. Mostly, studies investigating associations between noise exposure and noise annoyance have used measured or modelled noise exposure levels [2,5,8,46,47,48].

In this study, noise sensitivity was a borderline significant positive predictor of high to extreme annoyance, but a strong predictor of moderate to extreme levels of annoyance (in the binary logistic sensitivity model). It has been reported that subjective noise exposure is predicted by noise sensitivity [42]. In our bivariate model, noise sensitivity showed strong association with reported noise levels. Multicollinearity between these associated predictors may have attenuated the modelled influence of noise sensitivity on annoyance. It can be cautiously argued that should noise sensitivity lower the threshold of noise annoyance, other circumstantial and personal factors may then contribute more strongly to increased noise annoyance.

Worry about environmental health risks was associated with noise annoyance. Concerns about environmental safety is reportedly more common in noise sensitive people than the general public. This relationship may cause the “worry about environmental health risk” variable to behave in a similar way as noise sensitivity in a predictive model for noise annoyance. Miedema made conclusions supporting this view from a pooled analysis of 28 data sets [49].

Knowledge of health risks associated with traffic noise exposure was strongly associated with noise annoyance in our model. Intimate knowledge of risks associated with a stressor can breed concerns which would accentuate a subject’s reaction to that stressor. Knowledge could influence personal disposition and attitudes, and consequently may condition subjective responses to a perceived risk factor. Negative attitudes to a noise source could induce negative response to noise, but the relationship could go either way [3,27,47]. Awareness of residential noise risks can result because noise sensitive persons delve further into knowledge of risks than non-sensitive persons [47]. Although this study did not specifically evaluate attitude as a formal variable, a variable of similar construct could be worry about environmental risk.

Individual preference for nature has been observed to have a positive gradient with noise annoyance [24,25,50]. Greenery and scenic landscapes are thought to exert a restorative influence on the mind, thus diluting the anxieties of daily life [51]. Putatively, nature-oriented individuals would prefer that the equilibrium of nature remains pristine. Heinonen-Guzejev had adduced the noise reaction of the Finnish population to the swift transition from a long-held sparsely-populated agrarian territory to a highly industrialized economy with commensurate urbanization trends [52].

### 4.3. Factors Associated with Noise Sensitivity

We found that the distribution of noise sensitivity (composite scores) in the study population followed a Gaussian-type distribution, indicating that, in the general population, there are people at all estimated levels of noise sensitivity and that no clear cut-off mark exists delineating non-sensitive and sensitive subpopulations. In a study where noise sensitivity was measures on a five-point scale, Shepherd *et al.* found that their respondents were spread across all levels of noise sensitivity, with no one indicating a zero-level noise sensitivity [9]. The prevalence of high to extreme noise sensitivity, as assessed using a single question, was consistent with a preceding study based on a 1988 survey conducted among 1495 Finnish adults in which noise sensitivity was measured on a five-point scale using a single item question [42,52]. Overall, high to extreme noise sensitivity was quite prevalent. However, we cannot discount the possibility of study participation being influenced by a self-selection of environmentally sensitive individuals into the survey [53,54,55].

Noise sensitivity regressed strongly on noise annoyance. It is not implausible that a possible “positive feed-back loop” exists in which noise sensitivity lowers the threshold for noise annoyance, and noise annoyance—so induced—aggravates noise sensitivity. This view was initially proposed by Job [56] and later supported by Paunovic [5] who suggested that extended noise annoyance could worsen moods and trigger depressive episodes thereby rendering exposed subjects further noise sensitive.

### 4.4. Role of Age and Sex

In the bivariate models, being female showed some evidence of an association with noise annoyance and strong evidence of association with noise sensitivity. Age was not associated with these outcomes in any model. This is in contrast to a prior publication that showed associations between age-group and noise annoyance on one hand, and noise sensitivity on the other hand [48]. Sex was neither associated with noise sensitivity nor annoyance in any of the multivariate models. An early report of the elusiveness of such associations was made in 1972 [48]. Similar to a reports by van Gerven, our sensitivity model for noise annoyance yielded a statistically significantly association between age (45–59 years) and moderate to extreme noise annoyance in bivariate regression [10]. This association disappeared in the model where the response variable was high to extreme annoyance.

Although sex regressed well on both outcomes in bivariate models, presumably, the effect of sex may have been dampened by simultaneous inclusion with other covariates in the model. The effect of sex may have been confounded by variables which showed a sex differential and were associated with noise annoyance and sensitivity in the models; for example, knowledgeable about health risks, worry about environmental risks and positive environmental attitude. Our finding is corroborated by Li [50] who explored the role of sex within a multivariate model on noise sensitivity and by Bluhm [54] who compared the standardized mean difference in noise annoyance between men and women. Nevertheless, it has been reported that being female predicted higher noise annoyance and noise sensitivity in multivariate models [25].

### 4.5. Strengths and Weaknesses

This study relied on data from a broad-based national survey in which respondents were drawn from urban and rural residential areas, and provides a representative sample for the Finnish adult population. We assessed road-traffic noise exposure using subjective ratings of exposure which, in our opinion, is a closer representation of respondents’ actual experience than instrumental measurement or modelled noise levels. Like many previous studies, we focused on residential exposure because people spend more time at home, which makes exposure contribution from the home environment more substantial than contributions from other settings for most people. Based on results from previous studies, we believe that noise exposure-scale ratings agree to a reasonable extent with instrumentally measured levels of noise [25,39,41], with the added advantage that self-rated noise exposure incorporates individual factors which could influence perception of noise but would be lost during instrumental determination [28,42,47,57]. This paper re-emphasizes the importance of attitudinal variables, subjective knowledge and perception of health risks associated with road-traffic noise and exhausts, and how these could influence annoyance and sensitivity. These factors are less commonly explored among predictors in noise-effects research, and could explain some of the variation in noise annoyance and sensitivity.

An obvious weakness in this study is that the assessment of perceived noise exposure did not facilitate partitioning into daytime, evening or nighttime exposure. It therefore impairs any effort to isolate annoyance pertaining to specific segments of the 24-h day. Also, the modifying influence of building insulation is not taken into account. Our objective was to account for the best representation of the overall experience of respondents in their residential settings and to gauge factors that could explain this experience. We do not therefore see these omissions as a setback. Another weakness is that the questionnaire did not scrutinize personal circumstances and mental states that could impact on respondents’ assessment of exposure and annoyance. Our belief is that population-wide differences in such modifiers will not be distributed in such a way as would significantly alter our result when considered.

## 5. Conclusions

In conclusion, four in five Finnish adults felt exposed to road-traffic noise in their residential environment, proportionally more than in the occupational environment. The population health risk was also considered substantial. However, road-traffic noise was perceived to be of lower health risk than exhausts. Annoyance from noise was also widespread within the study population. These findings emphasise that road-traffic noise is a problem even in less populated, quieter societies.

High levels of noise annoyance were explained in part by the high perception of exposure, and noise sensitivity. No distinct noise sensitive subgroup could be identified. Only a low percentage of the variation in individual noise sensitivity could be explained; high sensitivity was associated with high noise annoyance. Knowledge of the health risks of noise and attitudinal variables, such as environmental attitudes and nature orientedness, seem to explain some of the variation in both noise annoyance and sensitivity. It would be informative to noise epidemiologists to further explore these variables in longitudinal studies, to elucidate their ramifications. That nature orientedness can predispose to noise annoyance and sensitivity should be of interest to town planners. There is a growing body of research which shows that green areas can ameliorate the impact of environmental stressors by providing a restorative influence on the mind.

## Appendix

**Table A1:** Questionnaire items on key variables.

**No.**	**Rating of Personal Noise Exposure**	**Response Scale**					
		No Exposure				Extreme Exposure	Unable to Answer
1	Please rate on a scale of 1–5 the typical extent of your exposure to road-traffic noise in your residential environment	1	2	3	4	5	8
2	Please rate on a scale of 1–5 the typical extent of your exposure to road-traffic noise in your occupational environment	1	2	3	4	5	8
	**Rating of Personal Traffic Exhausts Exposure**	No Exposure				Extreme Exposure	Unable to Answer
3	Please rate on a scale of 1–5 the typical extent of your exposure to road-traffic exhausts in your residential environment	1	2	3	4	5	8
4	Please rate on a scale of 1–5 the typical extent of your exposure to road-traffic exhausts in your occupational environment	1	2	3	4	5	8
	**Noise Annoyance**	No Disturbance				Extreme Disturbance	Unable to Answer
5	Are you disturbed in some way by road-traffic noise?	1	2	3	4	5	8
6	Are you disturbed in some way by road traffic exhaust?	1	2	3	4	5	8
	**Developing Symptoms**	Not at All				Very Much	Unable to Answer
7	Does road-traffic noise usually cause you to experience some kind of symptoms, for example, feeling ill, headaches, respiratory symptoms, eye irritation?	1	2	3	4	5	8
8	Does road-traffic exhaust usually cause you to experience some kind of symptoms, for example feeling ill, headaches, respiratory symptoms, eye irritation?	1	2	3	4	5	8
**No.**	**Perception of Health Risk**	**Response Scale**					
		No Risk				Extreme Risk	Unable to Answer
9	By your rating, how big a health risk is road-traffic noise to you?	1	2	3	4	5	8
10	By your rating, how big a health risk is road-traffic exhaust to you?	1	2	3	4	5	8
11	By your rating, how big a health risk is road-traffic noise to the general Finnish population?	1	2	3	4	5	8
12	By your rating, how big a health risk is road-traffic exhaust to the general Finnish population?	1	2	3	4	5	8
	**Personal worry**	Not Worried				Extremely Worried	Unable to Answer
13	In general, how worried are you about the health risks posed to you and your family by your residential environment?	1	2	3	4	5	8
	**Assessment of Personal Knowledge**	Nothing	Little	Some	A Lot	Very Much	
14	In your opinion, how much do you know about health risks associated with road-traffic noise?	1	2	3	4	5	
15	In your opinion, how much do you know about health risks associated with road-traffic exhausts?	1	2	3	4	5	
	**Environmental Attitudes**	Strongly Agree	Agree	Neutral	Disagree	Strongly Disagree	Unable to Answer
16	People needlessly worry that developmental activities cause damage to the environment	1	2	3	4	5	8
17	There are more important things in life than environmental protection	1	2	3	4	5	8
18	Many arguments regarding environmental threats are exaggerated	1	2	3	4	5	8
**No.**	**Nature Orientedness**	**Response Scale**					
		Strongly Agree	Agree	Neutral	Disagree	Strongly Disagree	Unable to Answer
19	I enjoy being in the city	1	2	3	4	5	8
20	I often feel distressed in a crowded and busy city	1	2	3	4	5	8
21	I feel that city centres are just the place for me	1	2	3	4	5	8
22	Green areas within cities do not fulfil my need to be in the nature	1	2	3	4	5	8
23	Sometimes I feel an urge to be in natural settings	1	2	3	4	5	8
24	I greatly appreciate areas with cafeterias, restaurants, museums, and theatres	1	2	3	4	5	8
25	I feel more comfortable in green areas and parks than in built environments	1	2	3	4	5	8
26	**Noise Sensitivity**	Strongly Agree	Agree	Neutral	Disagree	Strongly Disagree	Unable to Answer
	I get irritated when my neighbours cause noise	1	2	3	4	5	8
27	I am good at concentrating whatever happens around me						
28	It is difficult for me relax in a noisy place	1	2	3	4	5	8
29	I am sensitive to noise	1	2	3	4	5	8

**Table A2:** The distribution of some explored covariates between the strata of noise annoyance (high to extreme and others).

**Variables**	**No Annoyance to Some Annoyance**			**High to Extreme Annoyance**		
	Number	Mean (Standard Deviation)	Range	Number	Mean (Standard Deviation)	Range
Perceived exposure to traffic noise at home	832	1.39 (0.60)	1–3	188	2.61 (0.65)	1–3
Worried about personal and family risks from home environment	810	1.50 (0.70)	1–3	186	2.11 (0.77)	1–3
Noise sensitivity	811	8.42 (3.40)	1–16	187	10.05 (3.47)	2–16
Environmental attitude	751	4.70 (2.76)	0–12	170	3.79 (2.89)	0–11
City-nature orientedness	809	17.43 (5.35)	0–28	181	18.33 (5.15)	5–28
Knowledge of health risk from Traffic noise exposure	824	1.89 (0.73)	1–3	185	2.26 (0.73)	1–3
**Variables**	**No Annoyance to Some Annoyance**			**High to Extreme Annoyance**		
	Number	%		Number	%	
Knowledge of health risk from Traffic noise exposure	no knowledge	271	33		31	17
	some knowledge	371	45		74	40
	much knowledge	182	22		80	43
